# Interocular anatomical and visual functional differences in pediatric patients with unilateral cataracts

**DOI:** 10.1186/s12886-016-0371-5

**Published:** 2016-11-03

**Authors:** Erping Long, Jingjing Chen, Zhenzhen Liu, Zhuoling Lin, Qianzhong Cao, Xiayin Zhang, Xiaoyan Li, Lixia Luo, Haotian Lin, Weirong Chen, Yizhi Liu

**Affiliations:** State Key Laboratory of Ophthalmology, Zhongshan Ophthalmic Center, Sun Yat-sen University, Guangzhou, Guangdong 510060 China

**Keywords:** Interocular difference, Pediatric cataracts, Unilateral cataracts, Anatomical, Visual acuity

## Abstract

**Background:**

Congenital cataracts are often complicated by anterior segment dysgenesis. This study aims to compare bilateral anterior segment parameters, macular thickness, and best-corrected visual acuity (BCVA) in pediatric cataract patients at 3 months after unilateral cataract extraction with intraocular lens implantation.

**Methods:**

Fifty-three pediatric patients with uncomplicated unilateral total cataracts were included. At 3 months post-surgery, bilateral corneal thickness at the thinnest location (CTTL), anterior chamber depth (ACD), and anterior chamber volume (ACV) were measured using Pentacam. Central macular thickness (CMT) was evaluated using spectral-domain optical coherence tomography. BCVA was measured by experienced optometrists concurrently. Descriptive statistics and bivariate corrections were performed to analyze the interocular differences in bilateral anatomic parameters and their relationships with BCVA.

**Results:**

For all 53 included patients (mean age 5.2 ± 2.3 years), the median BCVA was 10/40 in the operated eyes and 40/40 in the contralateral eyes, which indicates a significant interocular difference. BCVA values in the contralateral eyes were significantly correlated with patient age at surgery, but this result differed for BCVA in the operated eyes. The Pentacam analysis revealed no significant interocular differences in bilateral CTTL and ACV, but significant differences were found for ACD.

**Conclusions:**

At 3 months after surgery, unilateral pediatric cataract patients exhibited no significant interocular differences in identified anatomical parameters (except for ACD), and these parameters were not significantly correlated with BCVA in bilateral eyes. Therefore, amblyopia, but not anatomical factors, might be the main cause of interocular visual functional differences in our study population.

**Trial registration:**

ClinicalTrial.gov, NCT02765230, 05/05/2016, retrospectively registered.

**Electronic supplementary material:**

The online version of this article (doi:10.1186/s12886-016-0371-5) contains supplementary material, which is available to authorized users.

## Background

Great advances have been made in managing pediatric cataracts during the last several decades; these advances have contributed to a decrease in the incidence of postoperative complications and improvement in visual outcomes [1, 2]. Early cataract removal and replacement with an intraocular lens (IOL) represent the most appropriate treatments to avoid irreversible amblyopia [3]. Recent studies have been directed toward understanding the factors associated with postoperative visual prognosis in pediatric patients with cataracts [4–7]. Although screening and timely surgical intervention play a key role in improved best-corrected visual acuity (BCVA) among pediatric patients with cataracts, determining the prognosis for an individual remains difficult, particularly for unilateral cataract [8–10].

Congenital cataracts are often reported to be complicated with anterior segment dysgenesis. However, with the exception of the cloudy lens in unilateral uncomplicated cataract patients, it remains unknown whether interocular differences exist in the developmental status of the anterior segment [11, 12]. Gochnauer et al. reported a trend wherein the absolute preoperative interocular axial length difference (IALD) predicted postoperative BCVA in pediatric patients with unilateral cataracts [13]. Their finding appears to be clinically important because it indicates the possibility of a correlation between ocular anatomy and visual function. However, the postoperative BCVA clinical outcomes of many pediatric patients without IALD still vary greatly [14].

Few studies have focused on the interocular differences in ocular anatomic parameters other than axial length in unilateral pediatric cataract after surgery, the significance of which for the possible interocular visual functional difference remains unclear. Therefore, we conducted this cross-sectional study to compare bilateral anterior segment parameters, macular thickness, and BCVA at 3 months after uncomplicated unilateral pediatric cataract extraction with primary IOL implantation and to explore any possible interocular differences and their possible relationships.

## Methods

### Patients

**Fig. 1:**
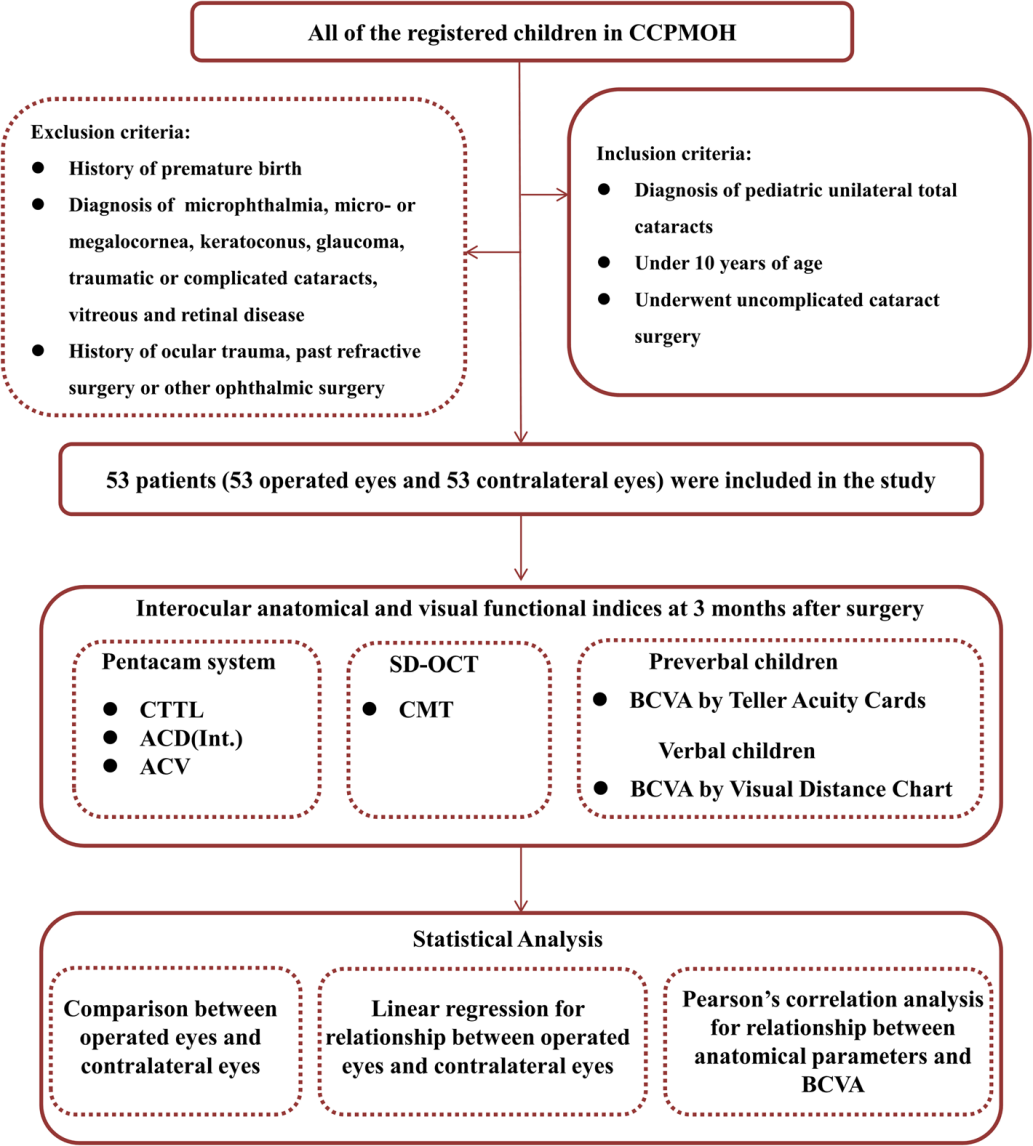
Flow chart of the patient selection and study protocol. (Notes: CCPMOH = Childhood Cataract Program of the Chinese Ministry of Health, CCTL = corneal thickness at the thinnest location, ACD (Int.) = anterior chamber depth measured from the endothelium, ACV = anterior chamber volume, SD-OCT = spectral-domain optical coherence tomography, CMT = central macular thickness, BCVA = best-corrected visual acuity)

This cross-sectional study was performed at Zhongshan Ophthalmic Center (ZOC), China’s largest eye hospital [15], which is the location of the Childhood Cataract Program of the Chinese Ministry of Health (CCPMOH) [16]. The CCPMOH is conducting a series of ongoing studies regarding the influence of early interventions on long-term outcomes of pediatric cataract treatment [17–19]. All of the registered children of CCPMOH were diagnosed by anterior segmental photography to confirm the morphology, location, and degree of cataract after pupil dilation [17]. A flow chart of patient selection and the study protocol is presented in Fig. 1.

### Ethical approval

The research protocol was approved by the Institutional Review Board/Ethics Committee of Sun Yat-sen University (Guangzhou, China). The tenets of the Declaration of Helsinki were followed throughout this study. To allow a confidential evaluation of the Pentacam system, optical coherence tomography (OCT), Teller visual acuity (VA) cards and visual distance chart use in this study, this trial was registered with the Clinical Research Internal Management System of ZOC as well as ClinicalTrials.gov (NCT02765230). The authors confirm that all ongoing trials and trials related to this study are registered.

### Inclusion and exclusion criteria

#### Inclusion criteria:

- Children with a diagnosis of pediatric unilateral total cataracts;
- Under 10 years of age;
- Underwent uncomplicated cataract surgery.

#### Exclusion criteria:

- History of premature birth;
- Diagnosis of microphthalmia, micro- or megalocornea, keratoconus, glaucoma, traumatic or complicated cataracts, or vitreous and retinal diseases;
- History of ocular trauma, past refractive surgery or other ophthalmic surgery.

### Intraoperative and postoperative procedures

All patients underwent uncomplicated cataract surgery by two experienced cataract surgeons (Y.Z.L. and W.R.C.). General anesthesia was administered prior to surgery. After a temporal clear corneal incision was made, DuoVisc and a soft-shell technique were used to reform and stabilize the anterior chamber and protect the corneal endothelium. A 5.5–6.0 mm central continuous curvilinear capsulorhexis was created using a bent 26-gauge disposable needle. Hydro-dissection was performed using a balanced salt solution, and a standard phacoemulsification was performed to completely remove the lens. A 3-piece monofocal acrylic IOL with a 6.5-mm optic diameter (Sensar AR40e, AMO, Inc. CA, USA) was placed via in-the-bag implantation following lens removal.

Postoperative topical therapy included the administration of 0.3 % tobramycin and 0.1 % dexamethasone eye drops (Tobradex, Alcon Laboratories, Inc, Texas, USA) four times per day and 0.3 % tobramycin and 0.1 % dexamethasone eye ointment (Tobradex, Alcon Laboratories, Inc, Texas, USA) every night for 1 month.

### Anterior segment parameters and the pentacam system

The Pentacam system (Oculus Inc., Wetzlar, Germany) was used for anterior segment parameter measurements at 3 months after surgery. Each examination was performed three times and reviewed by two observers to improve repeatability. At 3 months after surgery, the measurement procedure using the Pentacam was performed according to directions provided by the Pentacam instruction manual in cooperative children [20], and special requirements were followed for uncooperative children (described below). The Pentacam is a rotating scheimpflug camera that provides 50 cross-sectional images within a few seconds and has the advantage of generating a three-dimensional model of the anterior segment with a series of valued parameters (e.g., corneal thickness, corneal refractive, corneal volume, anterior chamber depth, anterior chamber angle, and anterior chamber volume) [21, 22]. Among the various anatomic parameters provided by the Pentacam, corneal thickness at the thinnest location (CTTL), anterior chamber depth (ACD), and anterior chamber volume (ACV) are the most representative parameters for the development status of the anterior segment [23]. Although corneal thickness is available for the entire cornea and can be measured at any point on the cornea by manually placing the cursor at that point, CTTL is defined as the corneal thickness at the corneal vertex in the absence of keratoconus. ACD, which is related to the anterior corneal apex position, is measured from the epithelium (external = Ext.) or endothelium (internal = Int.) to the anterior surface of the lens capsule or IOL. Therefore, the external ACD is the sum of the internal ACD and CTTL. In this study, we chose to report the internal ACD.

ACV is calculated as a solid bounded by the posterior surface of the cornea (12.0 mm around the corneal vertex), iris, and lens using integral calculus. The CTTL, ACD (Int.), and ACV are reported in the summary information for the Pentacam.

### Macular thickness and OCT

We used spectral-domain OCT (iTVue SD-OCT; Optovue Inc. Fremont, CA, U.S.A.) to evaluate the bilateral central macular thickness (CMT) of the included children with unilateral congenital cataract at 3 months after surgery [24, 25]. The macula was divided into three concentric circles with radii of 1, 3 and 6 mm, separately. The central disc is referred to as the fovea, and the inner and outer rings were equally segmented into four quadrants (i.e., superior, nasal, inferior, and temporal). The instrument calculated the macular thickness and summarized the thickness results corresponding to the Early Treatment Diabetic Retinopathy Study (ETDRS). Pupil dilation was not a mandatory requirement for CMT detection. Each subject’s eye focused on an internal fixation target without blinking and eye movement. Good compliance was noted while the scan was performed. However, for the children requiring examination under anesthesia, the procedure varied (described below). Five scans were oversampled, and the average for a cross-section was displayed.

### VA evaluation

For preverbal children, a complete set of Teller VA Cards (Stereo Optical Company, Inc., IL, USA) was used to measure the monocular grating acuity of the operated and contralateral eyes. The set consisted of 15 cards with gratings ranging in spatial frequency from 0.32 to 38 cycles/cm in half-octave steps as well as a low vision card and a blank gray card. The infant was assessed using the standard procedure of the operation manual [26, 27]. For verbal children, BCVA was measured and recorded using a LEA Symbols 13-Line Translucent Distance Chart (Good-Lite Co., IL, USA) according to the standard procedure. To minimize the bias of BCVA in both eyes, the examiner was blinded to the operated eyes, and the testing eyes were randomized across children. All children were assessed with good compliance for BCVA in both eyes in decimal fractions at 3 months after surgery, and the results were translated into Snellen BCVA and LogMar BCVA for analysis [28].

### Special requirements for uncooperative children

Because infants and young children have poor compliance with Pentacam and OCT examinations, we developed techniques and equipment specifically for pediatric ophthalmic examinations [17–19]. We performed examinations immediately after administering oral chloral hydrate, a retention enema with chloral hydrate as a sleep aid, or intranasal dexmedetomidine to children who were uncooperative for regular examinations in the clinic. This sleep-aid administration strategy has been preliminarily proven to be safe [29] and has been approved by the Institutional Review Board/Ethics Committee of Sun Yat-sen University. Briefly, the sleeping children were placed in the right posture. Upon assistance from the parents, the children’s heads were placed in positions suitable for Pentacam and OCT examinations with our designed device. An assistant helped to softly open the children’s eyelids [18]. More details can be found in our completed clinical trial (NCT02077712) regarding the sedation of pediatric cataract patients who were uncooperative during regular examinations at the clinic [30]. Three experienced pediatric ophthalmologists (H.T.L., J.J.C. and Z.L.L.) performed all examinations according to our study protocols.

### Statistical analysis

Data for different anatomical parameters measured by Pentacam examinations and OCT were entered into a Microsoft Excel spreadsheet (Microsoft Corporation, Redmond, WA, USA). Agreement between the readings obtained by the two observers was calculated for each eye of each participant using Bland-Altman limits of agreement. All measurements were determined to have a normal distribution. The results are expressed as the mean ±1 standard deviation (SD). An independent-sample-test was used to assess differences by sex, and a paired t-test was used to assess interocular differences. A bivariate correlation test (Pearson’s) was used to assess the relationship between different anatomical parameters (i.e., CTTL, ACD, ACV, and CMT) and a functional parameter (i.e., BCVA). All statistical tests were two-tailed, and a p-value below 0.05 was considered statistically significant. All statistical analyses were performed using SPSS software, version 17 (SPSS Inc., Chicago, IL, USA).

## Results

### Study population

A total of 53 registered patients (53 operated eyes and 53 contralateral eyes) treated at CCPMOH were included. All patients were Han Chinese. Thirty-six patients (67.9 %) were males. The average age at surgery was 5.2 ± 2.3 years, and no differences in age distributions by sex were noted. The right eye was involved in 23 (43.4 %) of the 53 cases with a unilateral total cataract. All included patients completed the Pentacam and OCT evaluations, but only 39 (39/53) postoperative eyes and 44 (44/53) contralateral eyes completed the BCVA function evaluation with credible results. Seven children had credible BCVA measured in only one eye, including 1 operated eye and 6 contralateral eyes. All the raw data supporting the conclusions of this article is available in Additional file 1, Raw data.

### Comparison between contralateral eyes and operated eyes

**Fig. 2:**
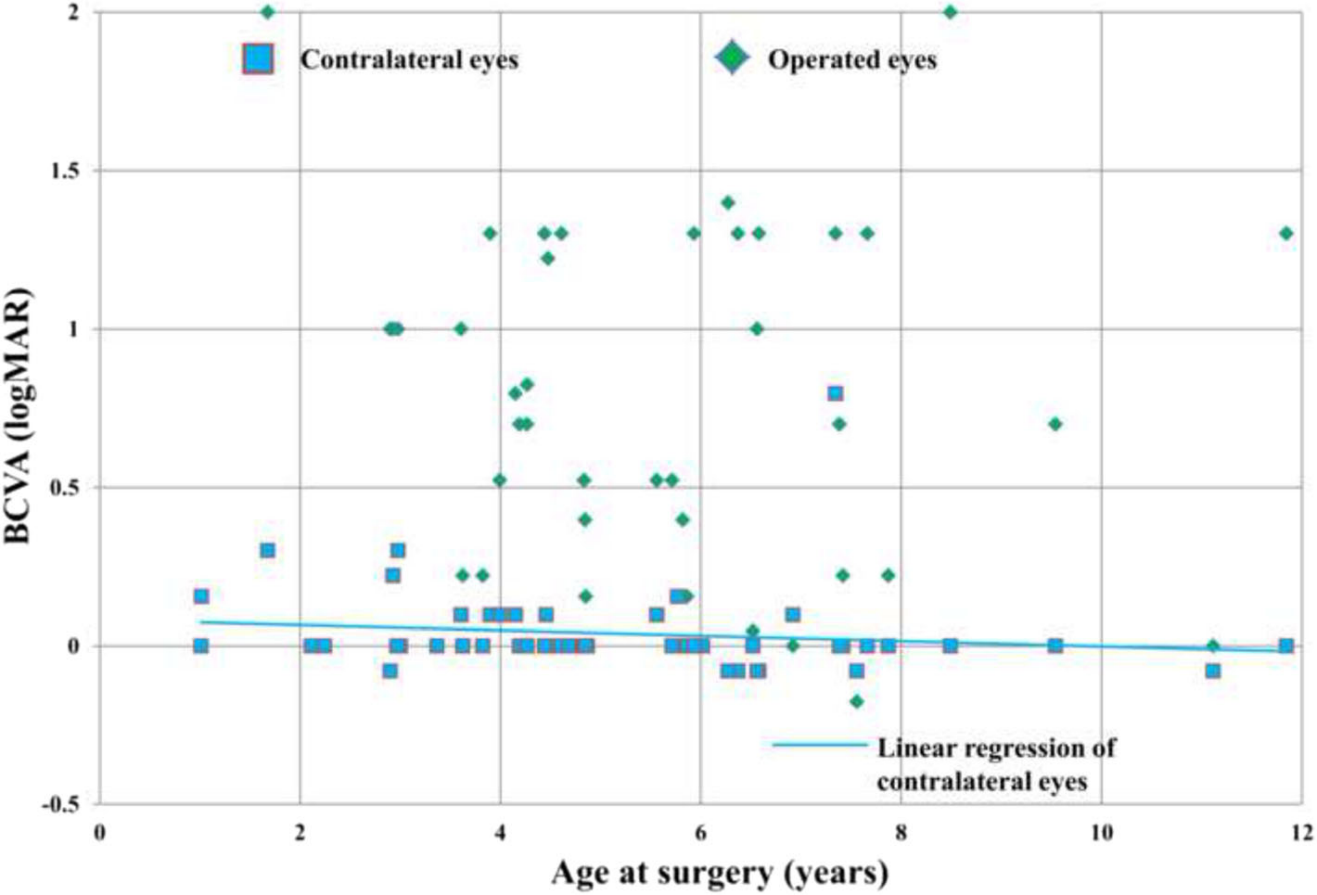
BCVA of contralateral and operated eyes at different age at surgery. Distribution of BCVAs between operated eyes and contralateral eyes was presented. The BCVA of operated eyes (*green rhombus*) is worse than those of contralateral eyes (*blue square*). BCVA values in the contralateral eyes were significantly correlated with patient age at surgery (Notes: BCVA = best-corrected visual acuity)

Except for one operated eye with a better BCVA than the contralateral eye, all operated eyes displayed worse BCVA values than the contralateral eyes (Fig. 2). The median BCVA was 10/40 in the operated group and 40/40 in the contralateral eye group, and a significant interocular difference was noted (*p* < 0.0001). Moreover, the age at surgery was significantly correlated with BCVA in the contralateral eye group (Pearson’s correlation = 0.356, *p* = 0.018) but not in the operated eye group (Pearson’s correlation = 0.129, *p* = 0.453).

In the Pentacam analysis, the mean CTTL, ACV, and ACD values in the operated eyes were compared with the contralateral eyes (Table 1). No statistically significant interocular differences in bilateral CTTL (*p* = 0.490) and ACV (*p* = 0.145) were noted, but the ACD values in the operated eyes were significantly increased compared with the contralateral eyes (*p* < 0.0001). In the OCT analysis, the mean CMT value in the operated eyes was compared with the contralateral eyes (Table 1), but the interocular difference in bilateral CMT was not significant (*p* = 0.708).

**Table 1:** The parameters and interocular differences in children with unilateral cataracts

Laterality	Numbers	CTTL (μm)	ACV (mm^3^)	ACD (mm)^c^	CMT (μm)
Operated	53	547.3 (58.9)^a^	164.3 (35.6)	3.7 (0.7)	228.2 (21.7)
Contralateral	53	542.7 (25.1)	157.1 (28.7)	2.9 (0.4)	229.2 (23.6)
Difference	N/A	4.6 [−8.7, 17.9]^b^	7.2 [−2.6, 17.0]	0.8 [0.6, 1.0]	−1.0 [−6.3, 4.3]

Footnotes: ^a^Mean (Std. Deviation); ^b^Mean [95 % Confidence Interval]; ^c^Significant (*p* < 0.0001); *CTTL* corneal thickness at thinnest location, *ACV* anterior chamber volume, *ACD* anterior chamber depth, *CMT* central macular thickness

### Linear regression of the relationship between operated eyes and contralateral eyes

**Fig. 3:**
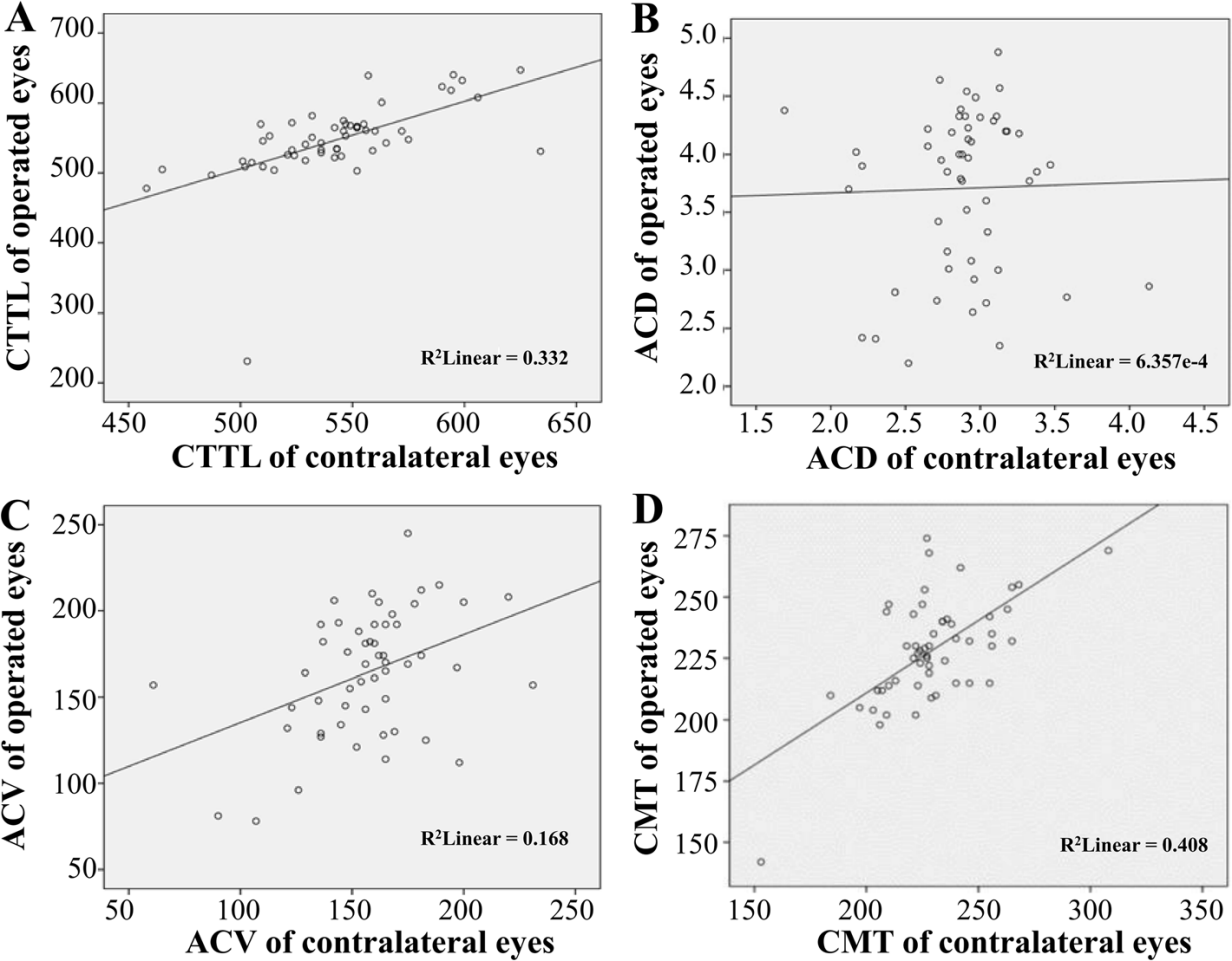
The relationships between the operated and contralateral eyes for CTTL, ACD, ACV, and CMT. Panel **a**, linear relationship for bilateral CTTL (*R* ^*2*^ = 0.332); Panel **b**, non-linear relationship for bilateral ACD (*R* ^*2*^ = 6.357e-4); Panel **c**, linear relationship for bilateral ACV (*R* ^*2*^ = 0.168); Panel **d**, linear relationship for bilateral CMT (*R* ^*2*^ = 0.408). (Notes: CCTL = corneal thickness at the thinnest location, ACD = anterior chamber depth, ACV = anterior chamber volume, CMT = central macular thickness, BCVA = best-corrected visual acuity)

To further analyze bilateral CTTL, ACV, ACD, and CMT, we used linear regression to predict the measurements of operated eyes in relation to contralateral eyes. We observed a good linear relationship for bilateral CTTL, ACV, and CMT but not bilateral ACD (Fig. 3).

### Pearson’s correlation analysis of the relationship between anatomical parameters and BCVA

**Fig. 4:**
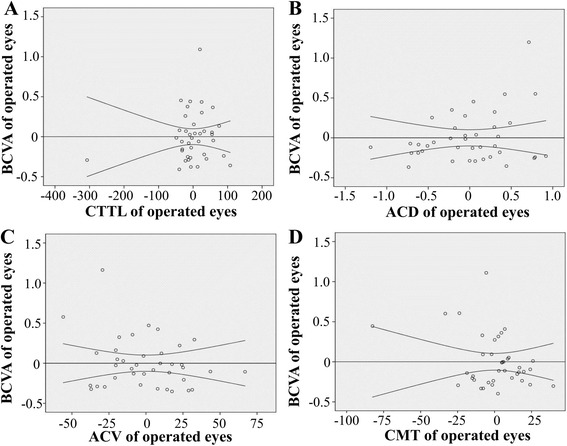
The relationships between BCVA and CTTL, ACD, ACV, or CMT in operated eyes. No significant correlations with BCVA were identified for CTTL, ACD, ACV, and CMT. Panel **a**, the relationship between CTTL and BCVA; Panel **b**, the relationship between ACD and BCVA; Panel **c**, the relationship between ACV and BCVA; Panel **d**, the relationship between CMT and BCVA. (Notes: CCTL = corneal thickness at the thinnest location, ACD = anterior chamber depth, ACV = anterior chamber volume, CMT = central macular thickness, BCVA = best-corrected visual acuity)

**Fig. 5:**
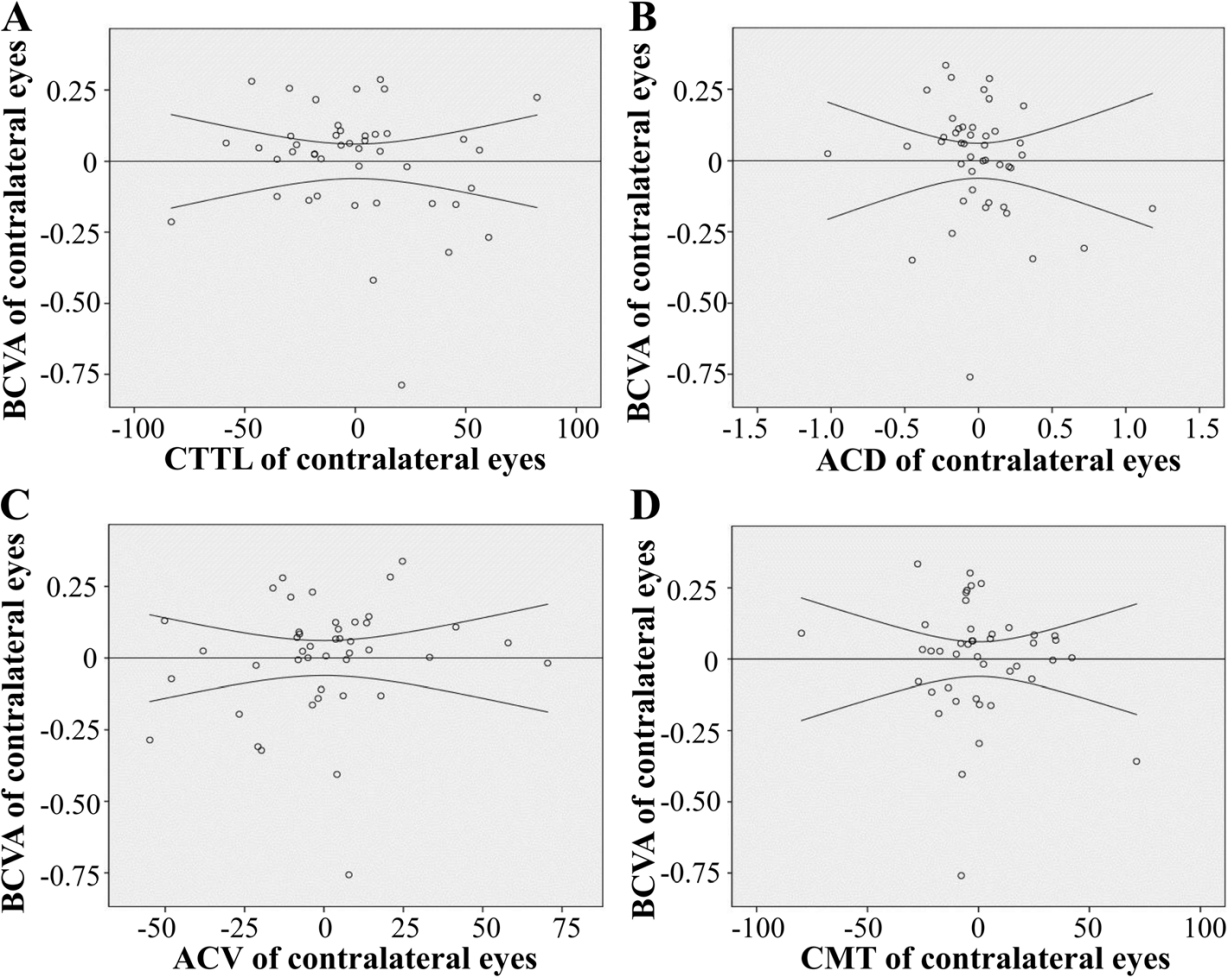
The relationships between BCVA and CTTL, ACD, ACV, or CMT in contralateral eyes. No significant correlations between BCVA and CTTL, ACD, ACV, or CMT were identified. Panel **a**, the relationship between CTTL and BCVA; Panel **b**, the relationship between ACD and BCVA; Panel **c**, the relationship between ACV and BCVA; Panel **d**, the relationship between CMT and BCVA. (Notes: CCTL = corneal thickness at the thinnest location, ACD = anterior chamber depth, ACV = anterior chamber volume, CMT = central macular thickness, BCVA = best-corrected visual acuity)

Another important and interesting previously unidentified relationship involves whether the analyzed anatomical parameters of CTTL, ACD, ACV, and CMT can predict the functional outcome of BCVA. Pearson’s correlation analysis was used to assess these relationships. In the operated eye, the correlations for CTTL, ACD, ACV, and CMT with BCVA were 0.084 (*p* = 0.611), 0.102 (*p* = 0.538), −0.050 (*p* = 0.761), and −0.273 (*p* = 0.092), respectively (Fig. 4). In the contralateral eye, the correlations were −0.241 (*p* = 0.114), −0.206 (*p* = 0.179), 0.059 (*p* = 0.703), and −0.163 (*p* = 0.290), respectively (Fig. 5).

## Discussion

In the present study, we analyzed bilateral ocular anatomical values by Pentacam examination and OCT in a population of term-born children with uncomplicated unilateral congenital/infantile cataract at 3 months after cataract extraction with primary IOL implantation. Cases of preterm birth and other ocular abnormalities (e.g., microphthalmia, micro- or megalocornea, keratoconus, glaucoma, and retinal disease) were excluded [13, 14, 31]. Thus, we were able to study a remarkably homogeneous population to explore the possible relationship between interocular differences of anatomy and function. We found no significant interocular differences in our identified anatomical parameters, with the exception of ACD, and all identified anatomical parameters were not significantly correlated with bilateral BCVA. Our present study helps demonstrate the significance of interocular differences in other ocular anatomic parameters in addition to axial length in unilateral pediatric cataracts and helps define the relationships among these differences with the BCVA of the operated/contralateral eyes.

In the present study, the Pentacam examination evaluates the entire anterior segment from the anterior corneal surface to the posterior lens surface using a rotating Scheimpflug camera [21, 22]. These non-contact measurements are finished in 2 s, and the technique obtains 12 to 50 single captures, which is convenient for children with limited compliance [18, 20]. Agreement was observed between all of the results of the anterior segment parameters obtained with the Pentacam and conventional methods. In the Pentacam analysis of our present study, the CTTL and ACV values were increased in the operated eyes compared with contralateral eyes without statistical significance. However, the ACD values were significantly increased in the operated eyes compared with the contralateral eyes; these differences were caused by the replacement of the natural lens with the artificial IOL and were not the basis for the interocular differences in BCVA [14]. However, we still cannot conclude that these identified interocular differences in the anterior segment caused the interocular differences in BCVA without further study.

Spectral-domain OCT is a commonly used commercial objective method for measuring retinal thickness, particularly CMT [25]. CMT is significantly correlated with VA in preterm children [32] or pediatric patients with diagnosed retinal diseases [33]. CMT is also correlated with BCVA in children with amblyopia based on long-term follow-ups and treatments [34, 35]. Kim et al. reported that spectral-domain OCT analysis of deprivational amblyopic eyes with unilateral pediatric cataracts demonstrated no significant differences in macular thicknesses compared with fellow non-amblyopic eyes and age-matched normal eyes [36]. In the present study, we also found that the mean CMT value in the operated eyes was reduced compared with the contralateral eyes, although the differences were not significant; these findings are consistent with the study by Kim et al.

To date, it has been difficult to predict the prognosis of BCVA among pediatric patients with uncomplicated unilateral cataract. In addition, the BCVA in the contralateral eyes are potentially significantly enhanced compared with the BCVA in the operated eyes in the uncomplicated unilateral cataract population [8, 10]. Our results have confirmed this finding. Moreover, in our study, the relationships between the identified anatomical parameters and the functional outcome of BCVA were not significantly correlated. All anterior segmental parameters (i.e., CTTL, ACD, and ACV) and CMT were not significantly correlated with BCVA in either the operated or contralateral eyes, which indicates that the significant interocular differences in BCVA might be caused by some unidentified factors. Although the reasons for our findings are complicated, deprivation amblyopia in the cataract eye and visual competitive inhibition of the contralateral eye should be considered [37, 38]. Therefore, it is crucial to conduct the long-term follow-up study with amblyopia treatment to investigate the therapy efficiency on our patients. Functional evaluations including functional magnetic resonance imaging [39–41] and visual electrophysiological techniques (for example, visual evoked potential) [42] have been widely used in clinical ophthalmology. We believe that these advanced techniques would aid in deciphering the real reasons for our findings and would also serve as a future research direction to optimize unilateral cataract treatment and improve prognoses.

Several limitations of this study should be considered. First, this study employs a cross-sectional design, which is prone to selection and measurement biases. However, we excluded preterm births and other pathologies accompanying eye conditions, thereby allowing us to precisely identify the type of patients to whom our results might be generalized (i.e., Chinese, term-born children with uncomplicated unilateral congenital/infantile cataracts, without other pathologic eye conditions). Second, all anatomical and functional indices were measured at 3 months post-surgery; therefore, we can only evaluate postoperative variation and draw conclusions about the development of interocular differences in anatomical indices and BCVA after cataract surgery. Despite the above limitations, to our knowledge, this study is the first to compare interocular differences in anterior segmental biometric and macular thickness values from a homogeneous series of infants and small children with unilateral congenital/infantile cataracts. In addition, it is also the first study to include statistical analysis of the relationships between BCVA outcomes and ocular anatomical parameters.

## Conclusions

In conclusion, our data on children with uncomplicated unilateral congenital/infantile cataracts at 3 months post-surgery did not reveal significant interocular differences in our identified anatomical parameters, with the exception of ACD. All analyzed anatomical parameters were not significantly correlated with bilateral BCVA. These results indicate that amblyopia, but not anatomical factors, might be the main cause of interocular visual functional differences in our study population. Our results may contribute to the knowledge of interocular anatomic differences in uncomplicated unilateral congenital/infantile cataracts and to the exploration of factors relating with operated BCVA in unilateral pediatric cataract patients. Interocular visual functional differences should be assessed to determine the causes, identify related factors and monitor the prognosis of amblyopia treatment.

## Additional file

Additional file 1: Raw data. (XLSX 13 kb)

## Abbreviations

ACD: Anterior chamber depth
ACV: Anterior chamber volume
BCVA: Best-corrected visual acuity
CCPMOH: The Childhood Cataract Program of the Chinese Ministry of Health
CMT: Central macular thickness
CTTL: Corneal thickness at the thinnest location
ETDRS: The early treatment diabetic retinopathy study
Ext.: External
IALD: Interocular axial length difference
Int.: Internal
IOL: Intraocular lens
OCT: Optical coherence tomography
SD: Standard deviation
VA: Visual acuity
ZOC: Zhongshan ophthalmic center
